# Atrial Fibrillation Screening in Nonmetropolitan Areas Using a Telehealth Surveillance System With an Embedded Cloud-Computing Algorithm: Prospective Pilot Study

**DOI:** 10.2196/mhealth.8290

**Published:** 2017-09-26

**Authors:** Ying-Hsien Chen, Chi-Sheng Hung, Ching-Chang Huang, Yu-Chien Hung, Juey-Jen Hwang, Yi-Lwun Ho

**Affiliations:** ^1^ Department of Internal Medicine National Taiwan University Hospital Taipei Taiwan; ^2^ Department of Medicine National Taiwan University Hospital JinShan Branch New Taipei Taiwan; ^3^ Department of Internal Medicine National Taiwan University Hospital Yun-Lin Branch Yun-Lin Taiwan

**Keywords:** atrial fibrillation, screen, cloud-computing algorithm, electrocardiography

## Abstract

**Background:**

Atrial fibrillation (AF) is a common form of arrhythmia that is associated with increased risk of stroke and mortality. Detecting AF before the first complication occurs is a recognized priority. No previous studies have examined the feasibility of undertaking AF screening using a telehealth surveillance system with an embedded cloud-computing algorithm; we address this issue in this study.

**Objective:**

The objective of this study was to evaluate the feasibility of AF screening in nonmetropolitan areas using a telehealth surveillance system with an embedded cloud-computing algorithm.

**Methods:**

We conducted a prospective AF screening study in a nonmetropolitan area using a single-lead electrocardiogram (ECG) recorder. All ECG measurements were reviewed on the telehealth surveillance system and interpreted by the cloud-computing algorithm and a cardiologist. The process of AF screening was evaluated with a satisfaction questionnaire.

**Results:**

Between March 11, 2016 and August 31, 2016, 967 ECGs were recorded from 922 residents in nonmetropolitan areas. A total of 22 (2.4%, 22/922) residents with AF were identified by the physician’s ECG interpretation, and only 0.2% (2/967) of ECGs contained significant artifacts. The novel cloud-computing algorithm for AF detection had a sensitivity of 95.5% (95% CI 77.2%-99.9%) and specificity of 97.7% (95% CI 96.5%-98.5%). The overall satisfaction score for the process of AF screening was 92.1%.

**Conclusions:**

AF screening in nonmetropolitan areas using a telehealth surveillance system with an embedded cloud-computing algorithm is feasible.

## Introduction

### Health Threats From Atrial Fibrillation

Atrial fibrillation (AF), a common form of sustained arrhythmia that has a significant impact on population health, is now a growing public health problem [1]. According to the Rotterdam Study, a large European population-based study, the overall prevalence of AF is 5.5% in a population of 55 years and older, rising from 0.7% in the age group of 55 to 59 years to 17.8% in those aged 85 years and older [2]. Meanwhile, in the ATRIA study from the United States, a cross-sectional study of adults aged 20 years or older, the overall prevalence of diagnosed AF was 0.95%, ranging from 0.1% among adults younger than 55 years to 9.0% in persons aged 80 years or older [3]. Both studies consistently demonstrated that the incidence of AF increased with age and was higher in men than in women. The number of patients with AF is likely to increase 2.5-fold during the next 50 years, reflecting the growing proportion of elderly individuals [3].

AF is considered a degenerative disease triggered by interactions with various substrate patterns, and it shares strong epidemiological associations with other cardiovascular diseases such as heart failure and coronary artery disease, rheumatic heart disease, hypertension, and diabetes. The incidence of AF varies depending on the population studied. The overall rate of incidence is 9.9 per 1000 person-years in a population older than 55 years according to the Rotterdam Study [2], whereas the Framingham Heart Study reports that the annual incidence is 0.5 per 1000 person-years [4]. AF is considered a risk factor for stroke [5,6] and congestive heart failure [7], and patients newly diagnosed with AF have a higher mortality risk, especially within the first 4 months of diagnosis [8]. There is a near 5-fold increase in the incidence of stroke when AF is present [6], and the annual risk of stroke ranges from 2% to 18% depending on other risk factors [9].

### Atrial Fibrillation Screening

Antithrombotic therapies, including vitamin K antagonists (VKA) [10,11] and nonvitamin K antagonist oral anticoagulants (NOAC) [12-15], reduce the risk of stroke in patients with AF. Currently, there is no effective way to prevent or cure AF and undiagnosed AF is common, especially in older populations and for patients with heart failure [16]. Previously, undiagnosed AF was found in 1.4% of those aged >65 years, which suggests that opportunistic screening for silent AF may be cost-effective in elderly populations [17]. The European Society of Cardiology (ESC) 2016 guidelines recommended conducting such screening by pulse taking or electrocardiogram (ECG) rhythm strips [18]. Currently, screening of older populations can be achieved through short-term ECG, pulse palpation [19], single-lead ECG [20-22], and blood pressure (BP) measurement with patented AF algorithm [23]. However, the sensitivity, accuracy, and accessibility of these modalities may affect the dissemination of AF screening, and the traditional 12-lead ECG has inherent limitations for its application to AF screening, especially in nonmetropolitan areas where the accessibility of health care is limited. The Telehealth Center of the National Taiwan University Hospital (NTUH) has conducted the fourth-generation telehealth service sin ce 2009 for patients with cardiovascular diseases [24-26]. By using ECG recorders (DigiO2 Cardio Care ECG recorder, DigiO2 International Co., Ltd), ECG measurement has become convenient and feasible at a distance from health care organizations. We conducted a prospective AF screening study in a nonmetropolitan area using a DigiO2 Cardio Care ECG recorder with a telesurveillance system embedded with a cloud-computing algorithm. The main purpose of the study was to evaluate the feasibility and accuracy of AF screening in nonmetropolitan areas.

## Methods

**Figure 1:**
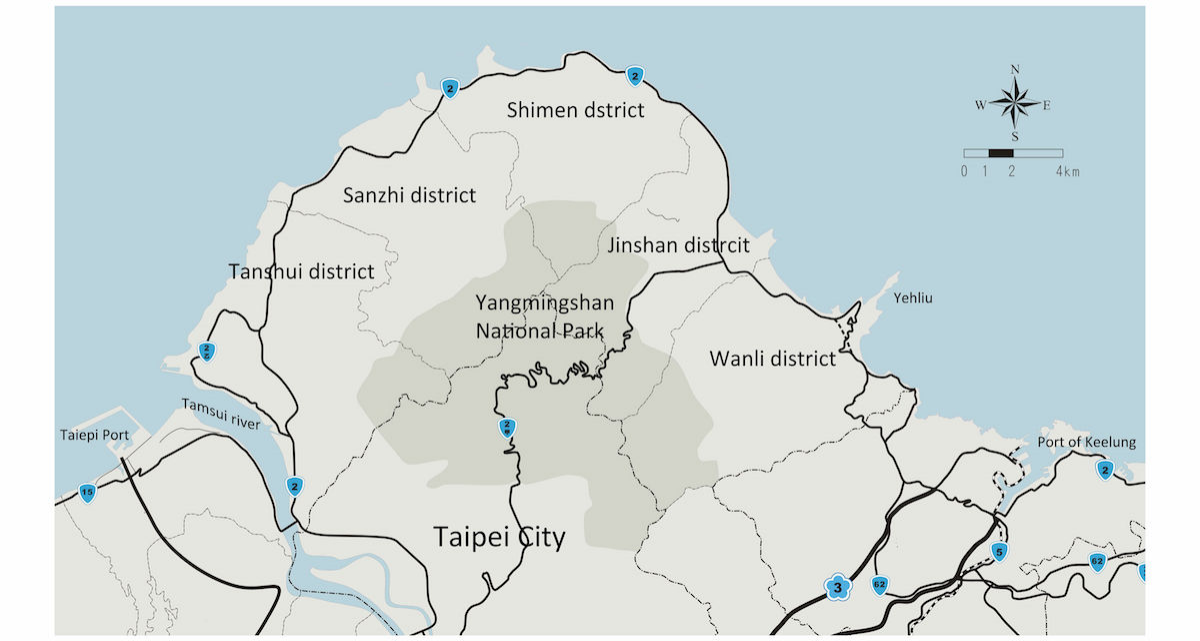
Map of nonmetropolitan area of JinShan, Wanli, Shimen and Sanzhi district.

The Taiwan ELEctroHEALTH (TELEHEALTH) study group conducted a prospective clinical study of AF screening in nonmetropolitan areas of Jinshan, Wanli, Shimen, and Sanzhi districts, New Taipei City, Taiwan. These areas were the northern coast of Taiwan with Yangmingshan National Park mountain barrier separating these areas from the metropolitan city (Taipei City) (Figure 1). The AF screening was conducted in the community during the advocacy activities held by the local health bureaus, various government agencies, and the National Taiwan University Hospital, Jinshan branch. A booth for AF screening was established. Local residents who attended the advocacy activity without active life-threatening medical conditions and aged older than 20 years were enrolled after obtaining their informed consent. Trained personnel would assist participants when performing ECG measurement according to the step-by-step instruction. The electrodes placement followed the manufacturer’s recommendation and in accordance with the American Heart Association recommendation [27]. Misplacement of electrodes can cause significant alteration to wave amplitudes or morphology, which may invalidate the use of recording [28]. All participants obtained an ECG measurement using the ECG recorder and completed a questionnaire regarding their individual health status, medical condition, and satisfaction toward the process of AF screening and handheld ECG measurement.

The measured ECGs were transmitted to a Web-based telesurveillance system at the Telehealth Center. An independent cardiologist performed the physician-based ECG interpretations. The computer-based ECG auto-interpretation was executed automatically according to the cloud-computing algorithm developed by the TELEHEALTH study group [29]. The institutional review board at NTUH approved the study protocol.

### Single-Lead ECG Recorder

**Figure 2:**
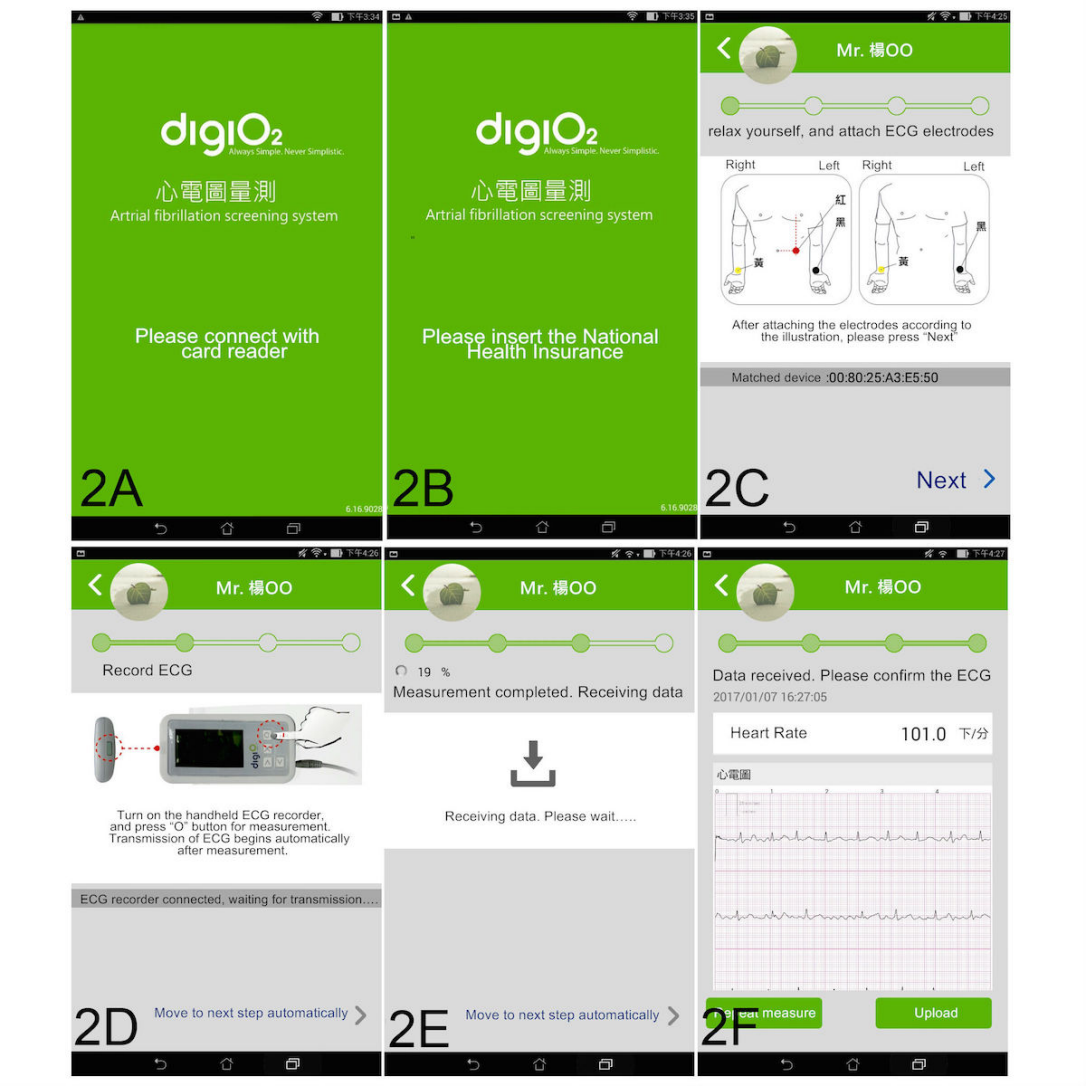
Steps in atrial fibrillation screening.

AF screening was performed in a stepwise manner under the assistance of trained personnel. After launching the Android-based AF screening application on an Android tablet, step-by-step instructions for the screening were displayed. Participants were instructed to connect the card reader to the Android tablet. If the card reader was connected to the tablet without launching the application first, the application would launch once the card reader was connected and proceed to the next step automatically. In the next step, participants were instructed to insert their identity-specific National Health Insurance Cards into the card reader to assess their personal information. After confirming their identity, a confirmation message was displayed, followed by an illustration showing how to position the ECG electrodes (Figure 2). The right arm limb lead (yellow) had to be placed anywhere between the right shoulder and right arm, the left arm limb lead (black) anywhere between the left shoulder and left arm, and the left leg lead (red) anywhere below the left torso and above the left ankle.

With the electrodes attached to their appropriate positions, the participants were instructed to press the measurement button, and the ECG recorder (DiGiO2 Cardio Care ECG Recorder) recorded a 15-second single-lead ECG. The measurements were transferred automatically from the ECG recorder to the tablet through a Bluetooth connection and could be explored instantaneously on the tablet through the application (Figure 2). The ECG was relayed from the tablet to the server at the Telehealth Center through a wireless local area network (WLAN) once the user had confirmed the upload. The ECG was then ready to be retrieved from the server at the Telehealth Center by the telesurveillance system, proceed to ECG auto-interpretation by the cloud-computing algorithm, and receive physician interpretation.

### Telesurveillance System

**Figure 3:**
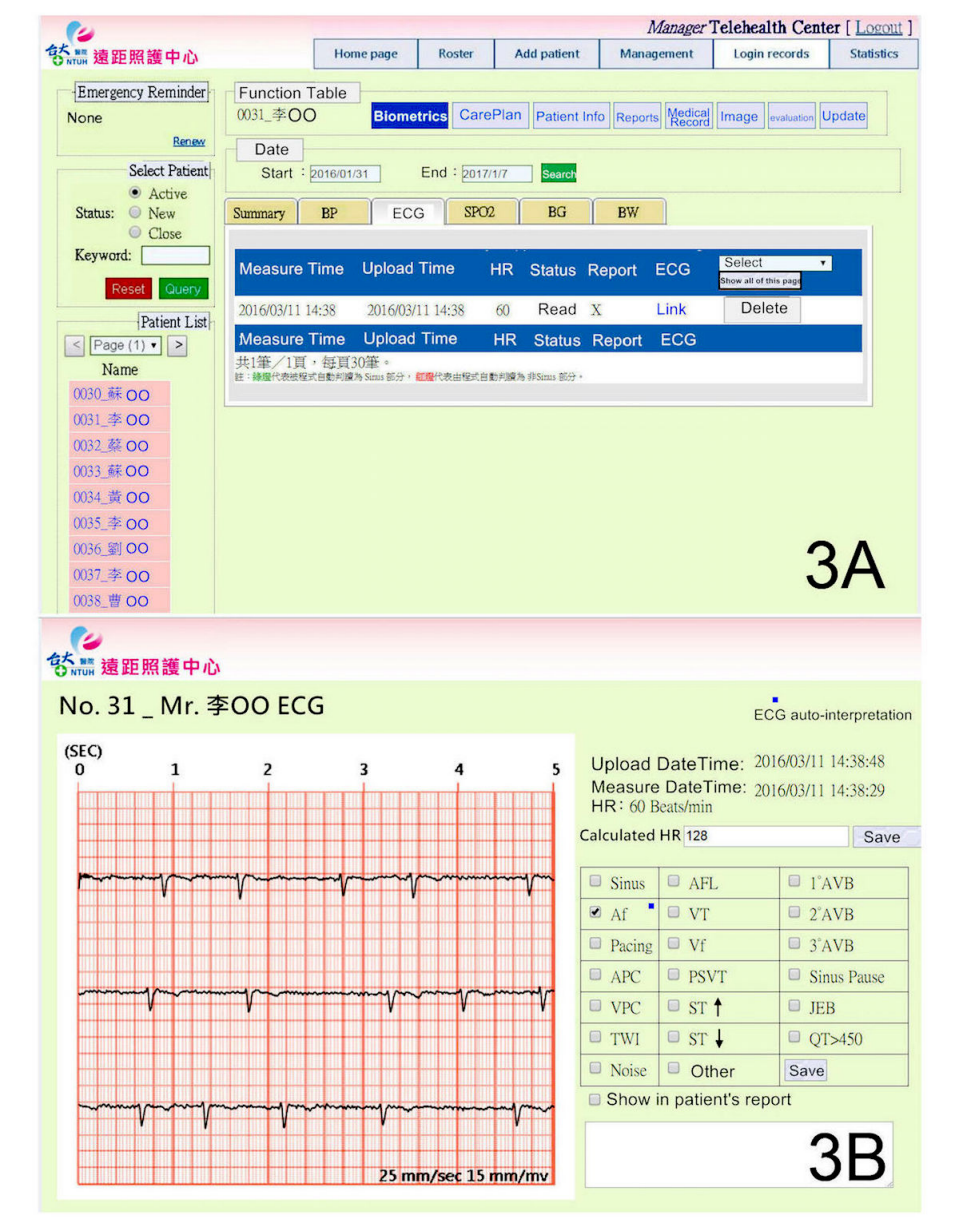
Tele-surveillance system and auto-interpretation by cloud-computing algorithm.

The telesurveillance system (Figure 3) is a Web-based platform developed by the Graduate Institute of Biomedical Electronics and Bioinformatics, National Taiwan University, Taiwan [29]. The telesurveillance system was operated under a service-oriented architecture framework with the Health Level Seven standard. Part of the function of the telesurveillance system includes exploring and reviewing biometric data, such as single-lead ECGs, BP, heart rate, and oximetry, and transferring the patient data to our Telehealth Center daily and on demand [26]. The physician’s ECG interpretation was also recorded on this Web-based platform.

### Cloud-Computing Algorithm

The design and algorithm of the Web-based ECG auto-interpretation were described in a previous study [29]. After removing baseline noise by finite impulse response filter, the key features of the ECG waveforms extracted were processed by support vector machine or rule-based processing to construct a classification model that can suggest diagnosis. A modified cloud-computing algorithm for determining AF was adopted during the study, where the detection of atrial premature complex (APC) or ventricular premature complex (VPC) was not included. Figure 3 demonstrates the result of ECG auto-interpretation by the cloud-computing algorithm on the telesurveillance system.

### ECG Quality Analysis and Artifacts Grading

**Figure 4:**
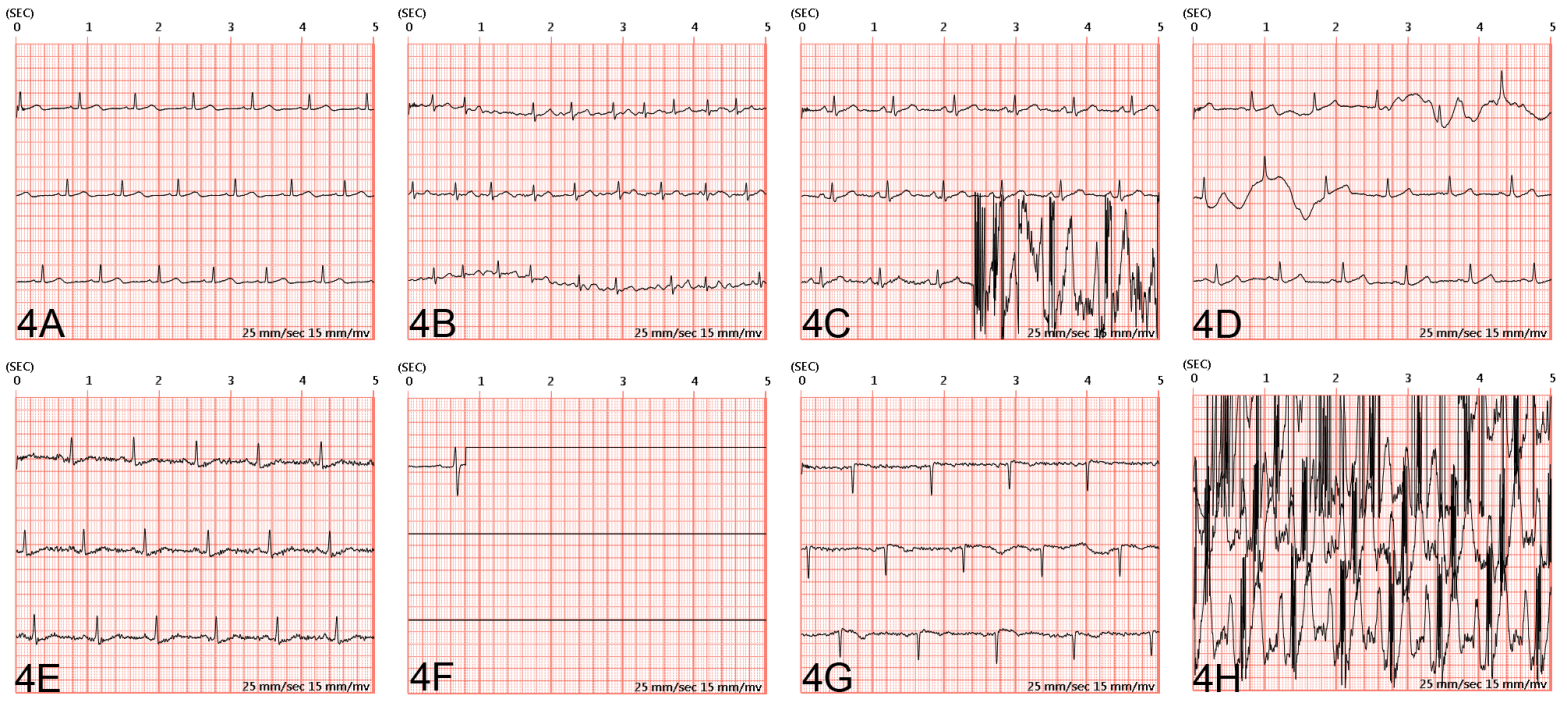
Illustrative electrocardiogram (ECG) and noise grading.

An independent cardiologist evaluated the ECG quality while performing physician ECG interpretation. The quality of ECG was categorized from grade 0 to grade 3 artifacts. A grade 0 artifact represents excellent image quality without artifacts. A grade 1 artifact represents an artifact percentage of <33% with recognizable P waves. A grade 2a artifact represents an artifact percentage of 33% to 66% or with a mild wandering baseline artifact with recognizable P waves. A grade 2b artifact represents an artifact percentage of >66% or with significant wandering baseline artifacts interfering with P wave recognition. A grade 3 artifact represents significant artifacts without recognizable P waves or QRS complexes. Figure 4 shows the illustrated examples of ECG artifacts in each categorization.

### Statistical Analysis

All continuous variables were expressed as mean (standard deviation [SD]) and categorical variables in numbers and percentages. Stata/SE 11.0 for Windows (StataCorp LP) was used for statistical analyses. The results of sensitivity, specificity, positive predictive value (PPV), and negative predictive value (NPV) were further stratified according to age.

## Results

Between March 11, 2016 and September 8, 2016, 967 ECGs were recorded from 922 residents (age: 58.1 [SD 15.0] years; aged >65 years: 426/922, 46.2%; male participants: 337/922, 36.6%) in Jinshan, Wanli, Shimen, and Sanzhi districts, New Taipei City, Taiwan, through community-based AF screening. Among those who received ECG measurements, 885 participants received a single ECG test, whereas 34 participants received two ECG tests, and 3 participants received three or more ECG tests. Among the 967 ECG records, 807 (807/967, 83.5%) were categorized as grade 0 artifacts, 124 (124/967, 12.8%) ECGs were categorized as grade 1 artifacts, 26 (26/967, 2.7%) ECGs had grade 2a artifacts, 8 (8/967, 0.8%) ECGs had grade 2b artifacts, and 2 (2/967, 0.2%) ECGs were classified as grade 3 artifacts.

The results of physician’s ECG interpretations demonstrated a sinus rhythm in 939 (939/967, 97.1%), including a sinus rhythm without ectopic beats in 907 patients (907/967, 93.8%), a sinus rhythm with APC in 15 patients (15/967, 1.6%), a sinus rhythm with VPC in 19 patients (19/967, 2.0%), and a sinus rhythm with both APC and VPC in 1 patient (1/967, 0.1%). There were 22 (22/967, 2.3%) AF rhythms, including AF rhythm with VPC in 3 (3/967, 0.3%). A paced rhythm in 1 (1/967, 0.1%) and a junctional rhythm in 3 (3/967, 0.3%) were identified by physician’s-determined ECG interpretations. Two ECGs (2/967, 0.2%) had no physician-determined ECG interpretations because of the presence of grade 3 ECG artifacts. The estimated prevalence of AF from our study population is 2.4% (22/922).

The results of the ECG auto-interpretation demonstrated no AF in 922 ECG measurements (92/967, 95.3%) and AF in 45 ECG measurements (45/967, 4.7%). After excluding ECG measurements with grade 3 artifacts, the overall performance for the AF screening cloud-computing algorithm had a sensitivity of 95.5% (95% CI 77.2%-99.9%) and a specificity of 97.7% (95% CI 96.5%-98.5%), with a PPV of 48.8% (95% CI 38.5%-59.3%), and an NPV value of 99.9% (95% CI 99.3%-100.0%) for detecting a disease prevalence (by ECG numbers) of 2.3% (22/965). When stratified to participants aged older than 65 years, the cloud-computing algorithm for AF screening had a sensitivity of 94.4% (95% CI 72.7%-99.9%) and a specificity of 96.4% (95% CI 94.2%-98.0%), with a PPV of 53.1% (95% CI 40.5%-65.4%) and an NPV of 99.8% (95% CI 98.4%-100.0%) for detecting a disease prevalence (by ECG numbers) of 4.1% (Table 1).

**Table 1:** Performance of the cloud-computing algorithm stratified according to age.

Result of cloud-computing algorithm	Age ≤65 years (n=526)	Age >65 years (n=439)	Overall (N=965)
Sensitivity, % (95% CI)	100.0 (95% CI 39.8%-100.0%)	94.4 (95% CI 72.7%-99.9%)	95.5 (95% CI 77.2%-99.9%)
Specificity, % (95% CI)	98.7 (95% CI 97.3%-99.5%)	96.4 (95% CI 94.2%-98.0%)	97.7 (95% CI 96.5%-98.5%)
Positive predictive value, % (95% CI)	36.4 (95% CI 21.5%-54.4%)	53.1 (95% CI 40.5%-65.4%)	48.8 (95% CI 38.5%-59.3%)
Negative predictive value, % (95% CI)	100.0	99.8 (95% CI 98.4%-100.0%)	99.9 (95% CI 99.3%-100.0%)
Disease prevalence (by ECG numbers), % (n/N)	0.8 (4/526)	4.1 (18/439)	2.3 (22/965)
Disease prevalence (by screening resident numbers), % (n/N)	0.8 (4/496)	4.2 (18/426)	2.4 (22/922)

### False Positive Analysis

A total of 22 false positive ECGs from the cloud-computing algorithm were identified. We analyzed the ECG quality and ECG characteristics. In addition, 16 (72.7%) ECG measurements had good image quality (grade 0 artifact), 3 (13.3%) had grade 1 artifacts, and another 3 (13.3%) had grade 2a artifacts. Other ECG features in these 22 false positives include a sinus rhythm with APC in 8 patients (36.4%), a sinus rhythm with VPC in 8 (36.4%), and a QRS complex voltage <0.5 mV in 9 (40.9%). There was only 1 false negative ECG from the cloud-computing algorithm.

### Satisfaction Questionnaire

In total, 825 satisfaction questionnaires were obtained from 922 participants, with an overall satisfaction score of 92.1%. Regarding AF screening convenience, 91.2% (752/825) of participants rated 4 or 5 on a scale of 1 to 5, and 89.5% (738/825) of the participants would recommend others to receive AF screening in the future.

## Discussion

### Principal Findings

We conducted a prospective AF screening study on nonmetropolitan areas through a single-lead ECG recorder and a telehealth surveillance system with an embedded cloud-computing algorithm. We compared the results obtained from ECG auto-interpretation by the cloud-computing algorithm and a cardiologist and found that the ECG recorder can obtain high-quality ECG images and that the ECG auto-interpretation by the cloud-computing algorithm for AF detection has a sensitivity of 95.5% and a specificity of 97.7%, with a relative low PPV of 48.8%. The overall satisfaction score for the process of AF screening was 92.1%.

With the prevalence and incidence of AF increasing with age, AF is a growing public health problem [1]. AF management has recently evolved to include high-risk patient identification via the CHA2DS2-VASc score, bleeding risk evaluation via the HAS-BLED score, and the SAMe-TT2R2 score for the initial selection of VKA or NOAC therapy [30]. The etiology for AF comprises complex pathophysiological changes in the atrium, including stretch-induced atrial fibrosis, hypocontractility, fatty infiltration, inflammation, remodeling, ischemia, and ion channel dysfunction.

There is no effective method to prevent AF, although some retrospective analyses from large randomized trials showed a lower incidence of new-onset AF in patients receiving angiotensin-converting enzyme inhibitors or angiotensin receptor blockers [31,32]. The early detection of AF and timely treatment before the first complications occur remain the best practice according to contemporary practices. According to the 2016 ESC guidelines, opportunistic screening for AF by pulse taking or the application of ECG rhythm strips in patients >65 years of age is now a class I indication [18]. However, traditional pulse palpation can be unreliable, and 12-lead ECG recordings can be cumbersome and might not readily be available or accessible to put into practice for AF screening practices. Other modalities such as AF detection during automated BP measurement, handheld ECG machines, mobile phone ECGs, and finger-probe instruments are thus under investigation for AF screening.

### Pulse Palpation

The classical sign of AF by pulse palpation is an irregular pulse. Sanmartin et al conducted a campaign for information and diagnosis of AF through pulse palpation. Among 1532 participants with a mean age of 73 (SD 7) years, 4.11% (63/1532) were identified with AF, including 1.11% (17/1532) with newly diagnosed AF [33]. Cooke G et al investigated three studies (2385 patients) that compared pulse palpation with ECG. The estimated sensitivity of pulse palpation ranged from 91% to 100% and specificity ranged from 70% to 77%. Pooled sensitivity was 94% (95% CI 84%-97%), and pooled specificity was 72% (95% CI 69%-75%). Given that pulse palpation has a high sensitivity but relatively low specificity for AF detection, it was considered a suitable tool for ruling out AF [34]. The diagnosis of AF still requires rhythm documentation using ECG [18,35].

### Twelve-Lead ECG

The typical pattern of AF on an ECG would be irregular RR intervals and no discernible, distinct P waves. Although economic analyses have concluded the cost-effectiveness of either annual screening [36] or opportunistic screening [17] by using a 12-lead ECG in those aged ≥65 years, the accessibility and higher cost for a 12-lead ECG may limit the dissemination of systemic screening. Other screening modalities are now under investigation for feasibility, accuracy, cost-effectiveness, and the potential to replace the 12-lead ECG.

### Screening for AF With Automated Blood Pressure Measurement

A specific algorithm for AF detection during automated BP measurement was developed and implemented in a novel oscillometric device (Microlife WatchBP Home-A). According to a meta-analysis composed of 6 studies (n=2332) performed by Verberk et al, the highest diagnostic accuracy for AF detection would be provided by using the Microlife BP monitor to take three sequential readings with at least two detecting AF, giving an estimated pooled sensitivity of 0.98 (95% CI 0.95-1.00) and specificity of 0.92 (95% CI 0.88-0.96) [23]. In 2013, the UK National Institute for Health and Care Excellence recommended this device for AF screening during routine office BP measurement in primary care for patients aged ≥65 years [37]. Although AF detection with routine-automated BP measurement could be a potential screening tool in the elderly people, it still requires confirmation by ECG [18,35].

### Handheld Single-Lead ECG Device

Desteghe et al evaluated the usability, accuracy, and cost-effectiveness of 2 handheld single-lead ECG devices (MyDiagnostick and AliveCor) for AF screening in a hospital population. The performance of the automated algorithm of each device was evaluated against a full 12-lead or 6-lead ECG recording. In the study, handheld recordings were not possible in 7% to 21.4% of hospital patients because they were unable to hold the devices properly. Both automated algorithms for each device had suboptimal sensitivity and specificity results. The sensitivity for MyDiagnostick was 81.8% to 89.5%, with a specificity of 94.2% to 95.7%. For AliveCor, the sensitivity was 54.5% to 78.9%, with a specificity of 97.5% to 97.9% [38].

For handheld DigiO2 Cardio Care ECG recorder, it is optional to use either dry contact electrodes or adhesive electrodes for ECG measurement; we preferred to use adhesive electrodes for ECG measurements to guarantee a better ECG quality and to test the accuracy of the cloud-computing algorithm. Therefore, use of adhesive electrodes for ECG measurement ensures that no participants are excluded from AF screening for being unable to hold the device. Moreover, even though an automated algorithm was embedded in the handheld single-lead ECG device in another study [38], our novel cloud-computing algorithm was embedded in a telesurveillance system, allowing a more comprehensive algorithm and a greater storage capacity for AF detection measurements.

### Finger-Probe Instruments

Lewis et al analyzed the application of a plethysmographic analysis of fingertip pulses in the detection of AF. A 12-lead ECG was recorded immediately for comparison when the finger probe was disconnected. The device detected all cases of AF (100% sensitivity), and a specificity of 91.9% (8.1% false positives) was obtained [39]. The finger probe may provide a potential tabletop instrument that allows for AF screening; however, it still requires a confirmatory ECG.

### Novel AF Detection Modalities

Photoplethysmography (PPG) is an optical method to measure changes in tissue blood volume caused by the pressure pulse. By placing a finger in contact with a mobile phone camera, the PPG waveform can be acquired through the light intensity reflected from a finger illuminated by the light-emitting diode mobile flash [40]. Chan et al [41] investigated the ability of PPG to diagnose AF in real-world situations. By using Cardiio Rhythm mobile app, the diagnostic sensitivity of the Cardiio Rhythm for AF detection was 92.9% (95% CI 77%-99%), which was higher than that of the AliveCor automated algorithm (71.4%; 95% CI 51%-87%). The specificities of Cardiio Rhythm and the AliveCor automated algorithm were comparable [41].

Nemati et al [42] proposed a noise-resistant machine learning approach to detect AF from noisy ambulatory PPG recorded from the wrist wearable technology. The preliminary result showed a sensitivity of 97% and specificity of 94% in 46 study subjects. Couderc et al proposed another novel technology for contactless detection of AF by using facial video recordings. The video plethysmographic signal acquired using a standard Web camera was extracted. A novel quantifier of pulse variability called the pulse harmonic strength was introduced to detect the presence of AF, which showed a 20% detection error rate [43]. Meanwhile, these new modalities still require confirmatory ECG for AF diagnosis.

### Strengths and Limitations

Although multiple modalities demonstrated a potential to be used in AF screening, some strengths and key features differentiated our study from others. First, we used adhesive electrodes to receive ECG signals to obtain the best ECG quality possible because we understand that the ECG is essential for AF diagnosis [18,35]. The ECG measurement first appears on site and can be explored through the application. Artifact ECGs can be identified before the final submission, and patients can repeat the ECG measurement when artifacts are presented. Although we use adhesive electrodes for ECG measurement, participants do not need to take off their clothes as with the traditional 12-lead ECG. By avoiding embarrassment and inconvenience brought by the removal of clothing, women should be more willing to receive AF screening. In fact, more women than men participated in our study (73.4%).

Second, our novel cloud-computing algorithm was embedded in a telesurveillance system, but not in the single-lead ECG devices per se, allowing a more comprehensive algorithm for AF detection. Moreover, the single-lead ECG recorder was used for ECG measurement only, and all measurements were transferred and stored in the cloud. There is only a requirement for temporary storage, which is a great advantage when AF screening is conducted in a large population where large amounts of ECG data are expected.

Third, the participant’s identification and ECG were matched and recorded electronically, which minimized the occurrence of error during data filing.

Fourth, the performance of AF screening through the ECG recorder and cloud-computing algorithm is satisfactory, with a high sensitivity (95.5%), specificity (97.7%), and NPV (99.9%). The result supports its use for AF screening in the future.

Fifth, the satisfaction questionnaire administered in our study received a high satisfaction score when graded by participants.

This study has several limitations. First, this is a single-arm study without comparison groups or randomization design. Second, our ECG measurements were not compared with the current gold standard 12-lead ECG. In this study, the AF screening was performed on a community basis, which made comparisons with a 12-lead ECG impossible. To compensate for this shortage, all single-lead ECGs were measured through adhesive electrodes to receive the highest ECG quality possible and make the use of a 12-lead ECG less necessary. Third, we measured a 15-second single-lead ECG during each AF screening by DigiO2 Cardio Care ECG recorder. Comparing with the other handheld ECG that measures 30 seconds ECG tracing, shorter ECG tracing in this study may raise the concern of diagnostic power for AF. The best method to compensate the shortage would be obtaining high-quality ECG for reference. In addition, we are able to preview the ECG instantaneously on the tablet before uploading to the server at the Telehealth Center. Repeat measurement was allowed if the ECG measurement was composed of artifacts. Fourth, although this study demonstrates that the ECG auto-interpretation by cloud-computing algorithm for AF detection has satisfactory sensitivity and specificity, the PPV of 48.8% is relatively low. A confirmatory examination is needed for the screened positive results from the ECG auto-interpretation by the cloud-computing algorithm. Fifth, an independent cardiologist performed all the physician-based ECG interpretations in the study. Potential interpretation error and bias may exist, as the interpretation of (single lead) ECG tracings can still vary between cardiologists. Most of the studies assigned 2 independent electrophysiologists for reviewing the ECGs [38,44]. Sixth, we did not evaluate the cost-effectiveness in this study. Potential expenditure could come from personnel expenses, adhesive electrodes, tablets, WLAN, and so on. Future studies for the cost-effectiveness should be performed before broadly applying our methodology for AF screening.

### Conclusions

It is feasible to conduct AF screening in nonmetropolitan areas using an ECG recorder with a telehealth surveillance system containing an embedded cloud-computing algorithm.

## Abbreviations

AF: atrial fibrillation
APC: atrial premature complex
BP: blood pressure
ECG: electrocardiogram
ESC: European Society of Cardiology
NOAC: nonvitamin K antagonist oral anticoagulants
NPV: negative predictive value
NTUH: National Taiwan University Hospital
PPG: photoplethysmography
PPV: positive predictive value
SD: standard deviation
TELEHEALTH: Taiwan ELEctroHEALTH
VKA: vitamin K antagonists
VPC: ventricular premature complex
WLAN: wireless local area network
